# Molecular characterization of plasma virome of hepatocellular carcinoma (HCC) patients

**DOI:** 10.1186/s13568-024-01696-2

**Published:** 2024-04-25

**Authors:** Niamat Ullah Khan, Asma Sadiq, Jadoon Khan, Nosheen Basharat, Zulfiqar Ul Hassan, Ijaz Ali, Tawaf Ali Shah, Mohammed Bourhia, Yousef A. Bin Jardan, Gezahign Fentahun Wondmie

**Affiliations:** 1 Molecular Virology Laboratory, Department of Biosciences, COMSATS University, Islamabad, Pakistan; 2 Department of Microbiology, University of Jhang, Punjab, Pakistan; 3 Department of Allied Health Sciences, Iqra University, Chak Shahzad Campus, Islamabad, Pakistan; 4 College of Agriculture Engineering and Food Science, Shandong University of Technology, Zibo, 255000 China; 5 Laboratory of Biotechnology and Natural Resources Valorization, Faculty of Sciences, Ibn Zohr University, Agadir, 80060 Morocco; 6 Department of Pharmaceutics, College of Pharmacy, King Saud University, P.O. Box 11451, Riyadh, Saudi Arabia; 7 Department of Biology, Bahir Dar University, P.O.Box 79, Bahir Dar, Ethiopia; 8 Center for Applied Mathematics and Bioinformatics (CAMB), Gulf University for Science and Technology, West Mishref, Kuwait

**Keywords:** Metagenomics, Anelloviruses, Viral etiologies, Hepatocellular carcinoma, Pathogenesis

## Abstract

Hepatocellular carcinoma (HCC) stands as the most common cancer type, arising from various causes, and responsible for a substantial number of cancer-related fatalities. Recent advancements in viral metagenomics have empowered scientists to delve into the intricate diversity of the virosphere, viral evolution, interactions between viruses and their hosts, and the identification of viral causes behind disease outbreaks, the development of specific symptoms, and their potential role in altering the host’s physiology. The present study had the objective of “Molecular Characterization of HBV, HCV, anelloviruses, CMV, SENV-D, SENV-H, HEV, and HPV viruses among individuals suffering from HCC.” A total of 381 HCC patients contributed 10 cc of blood each for this study. The research encompassed the assessment of tumor markers, followed by molecular characterization of HBV, HCV, Anelloviruses (TTV, TTMV, and TTMDV), SENV-H and SENV-D viruses, HEV, CMV, and HPV, as well as histopathological examinations. The outcomes of this study revealed that majority of the HCC patients 72.4% (276/381) were male as compared to females. HCV infection, at 76.4% (291 out of 381), exhibited a significant association (*p* < 0.05) with HCC. Most patients displayed singular lesions in the liver, with Child Pugh Score Type B being the predominant finding in 45.2% of cases. Plasma virome analysis indicated the prevalence of TTMDV (75%), followed by TTMV (70%) and TTV (42.1%) among anelloviruses in HCC patients. Similarly, SENV-H (52%) was followed by SENV-D (20%), with co-infections at 15%. The presence of CMV and HEV among the HCC patients was recorded 5% each however 3.5% of the patients showed the presence of HPV. In conclusion, this study underscores that HCC patients serve as reservoirs for various pathogenic and non-pathogenic viruses, potentially contributing to the development, progression, and severity of the disease.

**Supplementary Information:**

The online version contains supplementary material available at 10.1186/s13568-024-01696-2.

## Introduction

Hepatocellular carcinoma (HCC) stands as a leading cause of tumor-related fatalities, accounting for 745,000 annual deaths among males. Additionally, there are 800,000 new cases reported each year, with a projected increase to one million cases annually after 2025. HCC is currently ranked as the sixth most prevalent cancer type (Lovet et al. 2021). Most of the live cancer (70-90%) are HCC (El-Serag 2012) whose rate of mortality is increasing among North American and European countries. Many factors, both genetic and environmental, such as hereditary hemochromatosis, obesity, diabetes, aflatoxin exposure, and excessive alcohol consumption, significantly contribute to the development of hepatocellular carcinoma (HCC) (Gomaa et al. 2008). Furthermore, a significant portion of cases of hepatocellular carcinoma (HCC), estimated to be between 60 and 70%, has been attributed to chronic infections of the hepatitis C virus (HCV) or hepatitis B virus (HBV) (Ferlay et al. 2015), with a global incidence rate of 16 cases per 100,000 individuals. However, our understanding of the relationship between HCC and the hepatitis D virus (HDV) remains limited (Romeo et al. 2018). Additionally, majority of the HCC (60-70%) have been linked with infection of chronic hepatitis C virus (HCV) or hepatitis B virus (HBV) with an incidence rate of 16/100,000 cases worldwide, however less is known about the association with hepatitis D virus (HDV) (El-Serag 2012).

The microbiome refers to the combined genetic material of the diverse microbial populations that inhabit the human body, including bacteria (bacteriome), viruses (virome), archaea (archaeome), and protozoa (parasitome) (Berg et al. 2020). These microorganisms exist in symbiosis with the human host, residing in different locations within the body such as the gastrointestinal system, skin, respiratory tract, genital organs, and oral cavity (Lloyd-Price et al. 2016). Because of their significant involvement in human physiology, metabolism, nutrition and immune system this organ exert a significant influence on human health (Ley et al. 2008). In recent progress within the realm of viral metagenomics, a notable breakthrough occurred in discerning the evolution of viruses and exploring the diversity present within the virosphere (Chow and Suttle 2015; Paez-Espino et al. 2016; Zhang et al. 2018; Shi et al. 2018). Furthermore, characterization of viruses that causes outbreaks of diseases or specific symptoms (Delwart 2012; Zhou et al. 2020; Grard et al. 2012; Edridge et al. 2019; Ma et al. 2021; Wang et al. 2019), virus-host interaction and their significant role in disease and physiology of the host (Hannigan et al. 2018; Norman et al. 2015; Legoff et al. 2017; Lang et al. 2020; Zhao et al. 2017; Li et al. 2019) are also explored significantly. Additionally, in human context, the impartial viral metagenomics study significantly improves the identification and characterization of the viral sequences present in both diseased and healthy individuals which is used in clinical settings and increasingly accepted (Wilson et al. 2019; Zanella et al. 2021; Moustafa et al. 2017).

The main focus of prior research studies on human virome targeted skin, respiratory tract and gut viruses(Minot et al. 2013; Nayfach et al. 2021; Handley 2016; Dodi et al. 2021; Choi et al. 2021; Oh et al. 2016), however, the plasma/blood virome are also equally important as it comprises on both pathogenic and non-pathogenic or commensal viruses (Moustafa et al. 2017). Numerous viral infections can disrupt or alter the composition of the plasma virome. For instance, contracting HIV-1 may result in an elevated viral load of HERV (human endogenous retrovirus) and anellovirus (Li et al. 2012, 2013). The changes in the virome, specific to each infection, appear to be connected to a weakened immune system, which contributes to the advancement of the disease. This makes it challenging for researchers to determine whether the viruses already present in one’s system affect their overall health and play a role in their immune response (Liu et al. 2021). On the other hand, the characterization of blood and plasma virome analysis is equally important. This characterization plays a crucial role in ensuring the safety of transfusions and in monitoring for the presence of pathogenic viruses that can potentially spread during organ transplants or blood transfusions, leading to severe complications in different individuals (Zanella et al. 2020, 2021). The advancement in the field of metagenomics revealed the novel virus diversity involved in human health with a new ecological perspective (French and Holmes 2020; Hsu et al. 2021; Koonin et al. 2021; Liang and Bushman 2021). The most frequently observed viruses present in human plasma belong to the anelloviridae family, and they are often detected in conjunction with other non-pathogenic viruses (such as TTMV and TTV) as well as pathogenic viruses (including HSV, HPV, HIV, and HEV). These co-infections could potentially impact the severity of Hepatocellular Carcinoma (HCC). In Pakistan, there has been a recent increase in HCC cases throughout the country, prompting the need to investigate the common plasma virome in HCC patients. Therefore, the current study was conducted with the objective of molecularly characterizing the plasma virome in patients with HCC associated with HBV-HCV and those without this association, across different stages of the disease.

## Methodology

### Study design and ethical approval

This descriptive study was conducted at Molecular Virology Laboratory, Department of Biosciences, COMSATS University, Islamabad in collaboration with DINAR (D.I Khan Nuclear Medicine and Radiotherapy) Hospital, Mufti Mehmood Teaching Hospital, and MARDAN Medical Complex from September 2018 to October 2019. The current study was ethically approved from institutional ethical committee of COMSATS University, Islamabad, Pakistan under Ethical Approval No. CUI-Reg/Notif. 2255/20/2691.

### Sample collection and processing

The current study used Open Epi calculator for sample size calculation keeping the prevalence rate of 10.7% of all types of cancer (95% confidence interval) for statistical assumptions of HCC as described earlier (Shehzad et al. 2019). As per calculation the required sample size was 145 patients. However, 381 patients were included in the study by using consecutive sampling method, irrespective of the gender and age of the patients. Patients with undergoing treatment or visiting for follow up were excluded from this study.

### Demographic characteristics and serological assessment

A detailed clinical history including age and gender of entire HCC patients were obtained through a comprehensive questionnaire. The status of patient’s performance were graded from 0 to 5 as per the criteria mentioned by WHO (Aljumah et al. 2016). Initial diagnosis of HCC was confirmed through α fetoprotein (AFP) > 400 followed by computed tomography (CT) scan of chest and liver. Milan’s criteria and BCLC was adopted for the diagnosis of various HCC stages (Llovet et al. 2008) including performance status of patients using ECOG criteria, size and spread of tumor (Oken et al. 1982). MELD criteria (Kamath and Kim 2007) and Child Pugh Score (Pugh et al. 1973; Child and Turcotte 1964) was used for the assessment of underlying chronic liver disease. Patients were divided in three different groups Child Class A (Child Pugh Score = 5–6), Class B (Child Pugh Score = 7–9) and Class C (Child Pugh score = 10–15) (Hafeez et al. 2020). Blood (05 ml) was taken under aseptic conditions in a clot activator gel tube and serum was separated by centrifugation at 5000 rpm for 03 min and was stored in aliquots at -20 °C.

### Nucleic acid extraction and cDNA Preparation

A total of 200 µl of Serum sample was used for nucleic acid (DNA/RNA) extraction by using Tianlong^™^ viral nucleic acid extraction kit (Suzhou Tianlong Bio-technology Co., LTD) in accordance with the manufacturer’s instructions. cDNA was prepared through RT-PCR followed by qualitative confirmation of entire viruses.

### Molecular characterization of HCV/HBV and prevalent viral genome

The quantitative analysis of HBV/HCV and other prevalent viruses (Anelloviruses, SEN virus, Herpes viruses, HPV) was performed. Primer sets were utilized to amplify the specific region confirming viral presence or absence (Table 1.). Initially a total of 25 µl of PCR mixture was prepared by mixing 5 µl DNA with 1 µl + 1 µl forward and reverse primer each, 5 µl commercially prepared master mix and 13 µl sterilized PCR water. For the nested PCR (SENV), in first round of PCR universal set of primers was used (S1, S2, S3 Tables). In second-round of PCR, 2 µl PCR product from round-one was used as template making total volume of 20 µl and specific primers were used which amplify specific region of viral genome. Initial denaturation was done at 95˚C for 5 min, following denaturation at 95˚C for 30 s, primer annealing at various temperature (Table 1) for 30 s, extension 72˚C for 45 Sect. (35 cycles), and final extension at 72˚C for 10 min.

### Statistical analysis

The entire collected data was fed into SPSS (ver. 16.00) and chi-square test was applied by keeping 95% CI and *P* < 0.05 considered as statistically significant.

## Results

### Demographic assessment of patients

Demographic and clinical observation showed that majority of HCC patients were males 72.4% (276/381) as compare to female 27.6% (105/381) where the mean age of the patients was 60.4 ± 8.67 years. Among the pathogenic hepatotropic viruses the prevalence of HCV 76.4% (291/381) was higher as compare to HBV 11.5% (44/381) in HCC patients. Majority of the patients restricted their physical activity, able to do sedentary work in their performance status 79% (301/381). Higher number of HCC patients was of Type B 45.2% (172/381) followed by Type C 25.2% (96/381) on the basis of Child Pugh score. Larger number of HCC patients 50.7% (193/381) presented BCLC stage B (intermediate), C or D (advanced) stage of HCC. For entire data see Table 1.

**Table 1:** Clinical and Demographic Characteristics of HCC Patients

Sr. No	Characteristics	N (%)
1	**Gender**	
2	Male	276/381 (72.4)
3	Female	105/381 (27.6)
**Age wise**		
5	18–30	70/381 (18.4)
6	31–45	86/381 (22.6)
7	46–60	121/381 (31.8)
8	61- above	104/381 (27.3)
**Etiology**		
10	HCV	291/381 (76.4)
11	HBV	44/381 (11.5)
12	HCV + HBV	17/381 (4.5)
13	NASH	13/381 (3.4)
14	Non HCV/HBV	16/381 (4.2)
**MELD score**		
16	< 10	204/381 (53.5)
17	11–20	153/381 (40.2)
18	21–30	19/381 (5)
19	31–40	05/381 (1.3)
**ECOG status**		
21	Fully active	17/381 (4.5)
22	Restricted physical activity, able to do sedentary work	301/381 (79)
23	Ambulatory capable of self-care, unable to carry out any work activities	46/381 (12.1)
24	Self-limited care on chair more than 50% working hours	12/381 (3.1)
25	Completely disable cannot carry on any self-care	05/381 (1.3)
**Child Pugh Score**		
27	A	113/381 (29.6)
28	B	172/381 (45.2)
29	C	96/381 (25.2)
**BCLC stage**		
31	0	13/381 (3.4)
32	A	57/381 (15)
33	B	193/381 (50.7)
34	C	106/381 (27.8)
35	D	12/381 (3.1)

### Serological analysis of hepatocellular carcinoma patients

Among the entire studied population, a total of 28.9% (110/381) has elevated level of AFP (400–1000) while 40.2% (153/381) of the patients presented 02 or more liver lesions where vascular lesion was observed in 31.2% (119/381) HCC patients as shown in the Table 2.

**Table 2:** Tumor Variables Analysis of Hepatocellular Carcinoma Patients

Sr. No	Characteristics	N (%)
1	**AFP level**	
2	< 400	211/381 (55.6)
3	400–1000	110/381 (28.9%)
4	> 1000	60/381 (15.7%)
5	**Tumor size**	
6	2 cm	49/381 (12.9)
7	3–5 cm	137/381 (35.9)
8	5–10 cm	131/381 (34.4)
9	> 10 cm	64/381 (16.8)
10	**Number of lesions**	
11	Single	164/381 (43)
12	2–3	153/381 (40.2)
13	4–5	57/381 (14.9)
14	> 5	07/381 (1.8)
15	**Vascular lesion**	
16	Present	119/381 (31.2)
17	Absent	262/381 (68.8)

### Serological and molecular detection of HBV/HCV in HCC patients

**Fig. 1:**
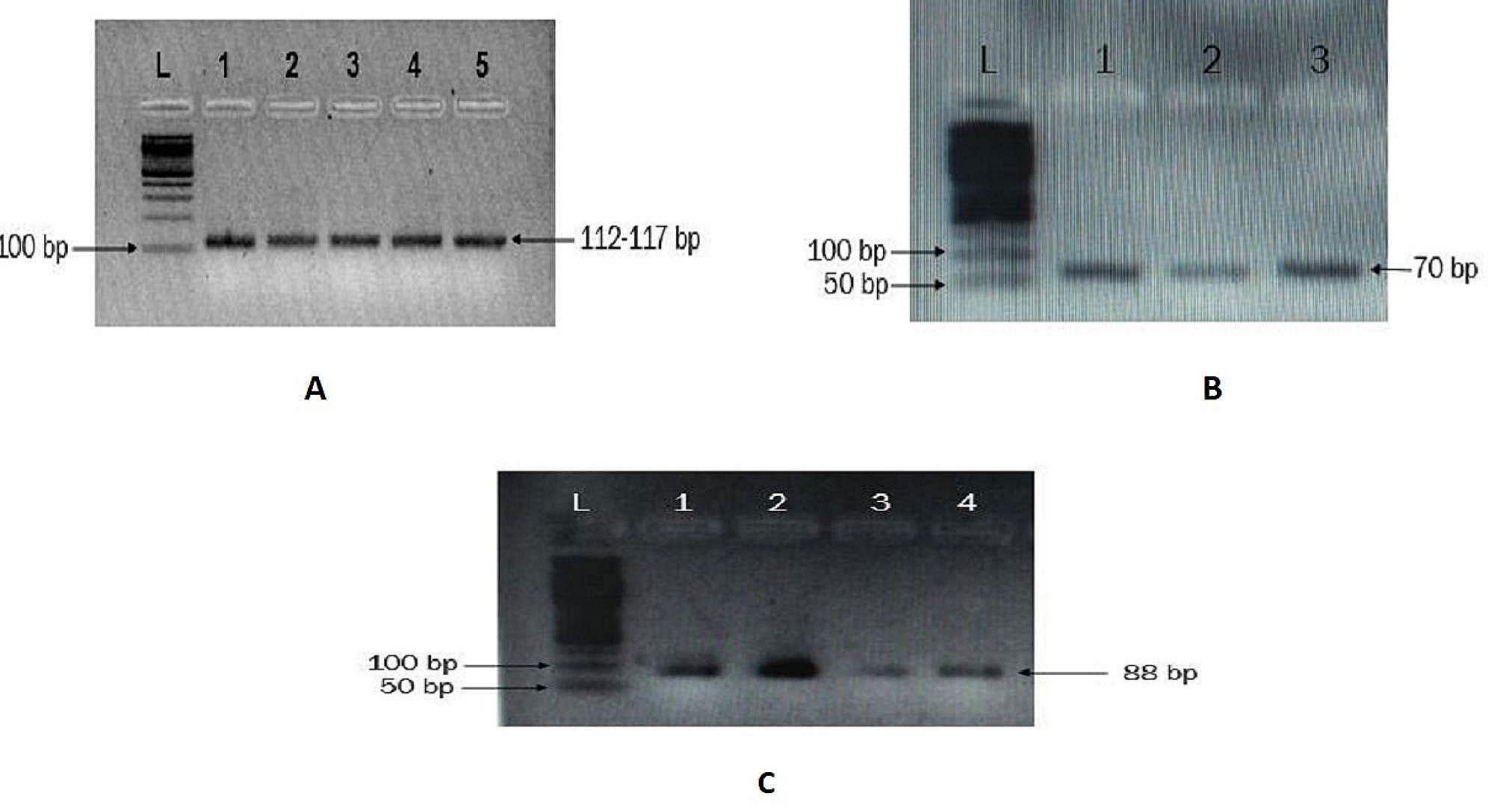
The first electropherogram **(A)** represent Torque teno virus [TTV], the second electropherogram **(B)** represent Torque teno mini virus [TTMV], and the third electropherogram **(C)** display transfusion transmitted midi virus [TTMDV] PCR product in 3.5% gel. Lane L denote the Molecular marker of 100 bp

Serological assessment showed that 80% (305/381) were positive for anti-HCV antibodies among which molecular confirmation observed 76.4% (291/381) subject’s positive for active HCV infection (Fig. 1). Serological analysis for HBV showed that 20% (78/381) of the samples were positive for anti-HBsAg which upon molecular confirmation 11.5% (44/381) were with active HBV infection as shown in the Fig. 1.

### Detection of SEN virus

**Fig. 2:**
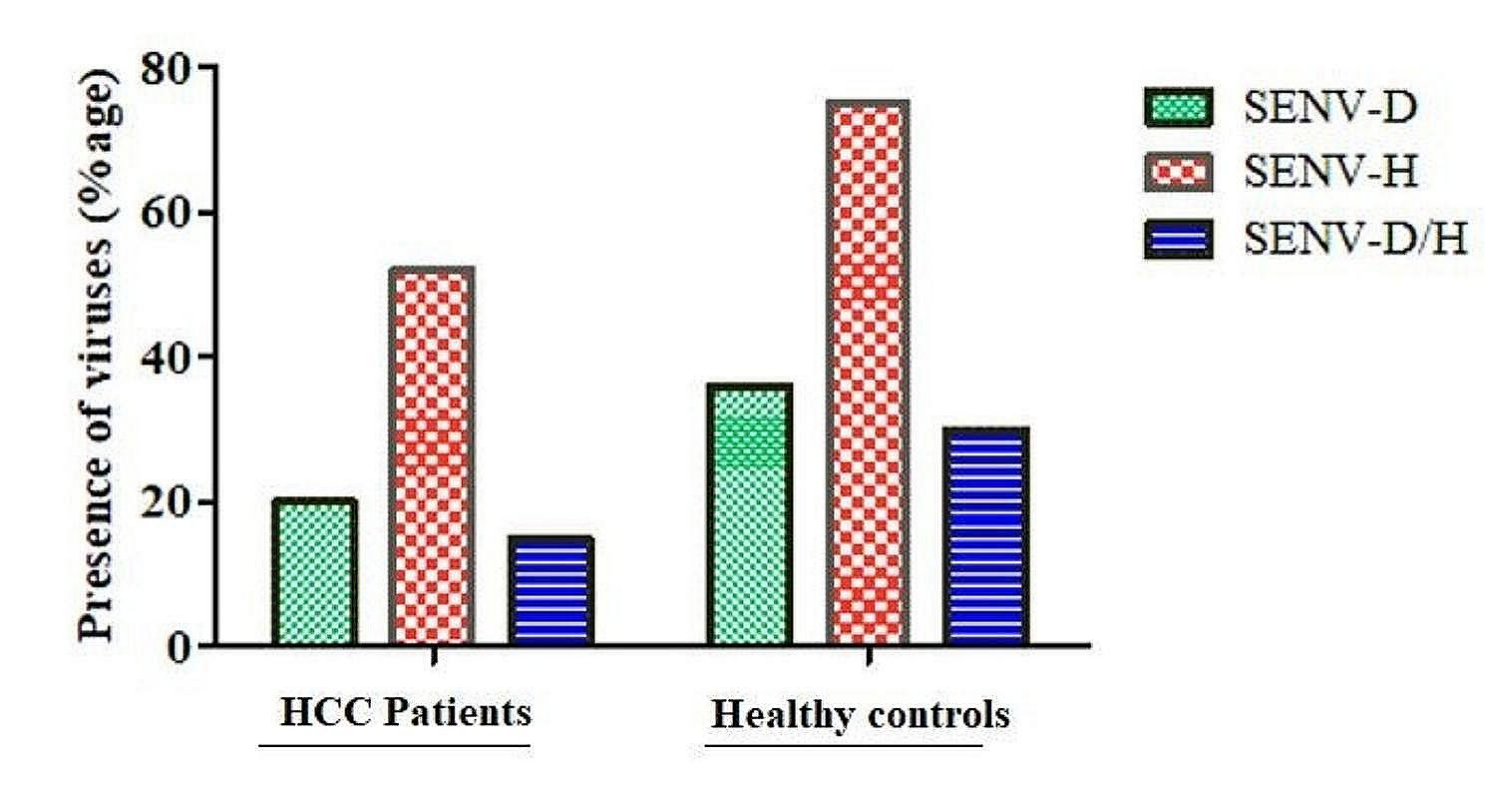
The graph shows the frequency of SENV in HCC patients and healthy blood donors. The SENV-D and SENV-H are 2 different variants of the SEN virus. Red dotted bar denotes only SENV-H variants, green dotted bar represents SENV-D, whereas the blue dotted bar refer to the ratio of both SENV-D/H variants

Among HCC infected individuals, 52% patients had a strong active infection of SENV-H while SENV-D was identified in 20% patients and 15% patients had co-infection of both SENV-D and H (Fig. 2). While in healthy control, the occurrence rate of SENV-D and SENV-H among healthy blood donors was 36% and 75% respectively, and 30% individual had co-infection of both SENV-D and SENV-H. The frequency of SEN virus types in healthy blood donors is given below Fig. 2.

### Detection of anelloviruses

Among Anelloviruses, TTMDV was detected in 75% followed by TTV was found in 42.1%.

**Fig. 3:**
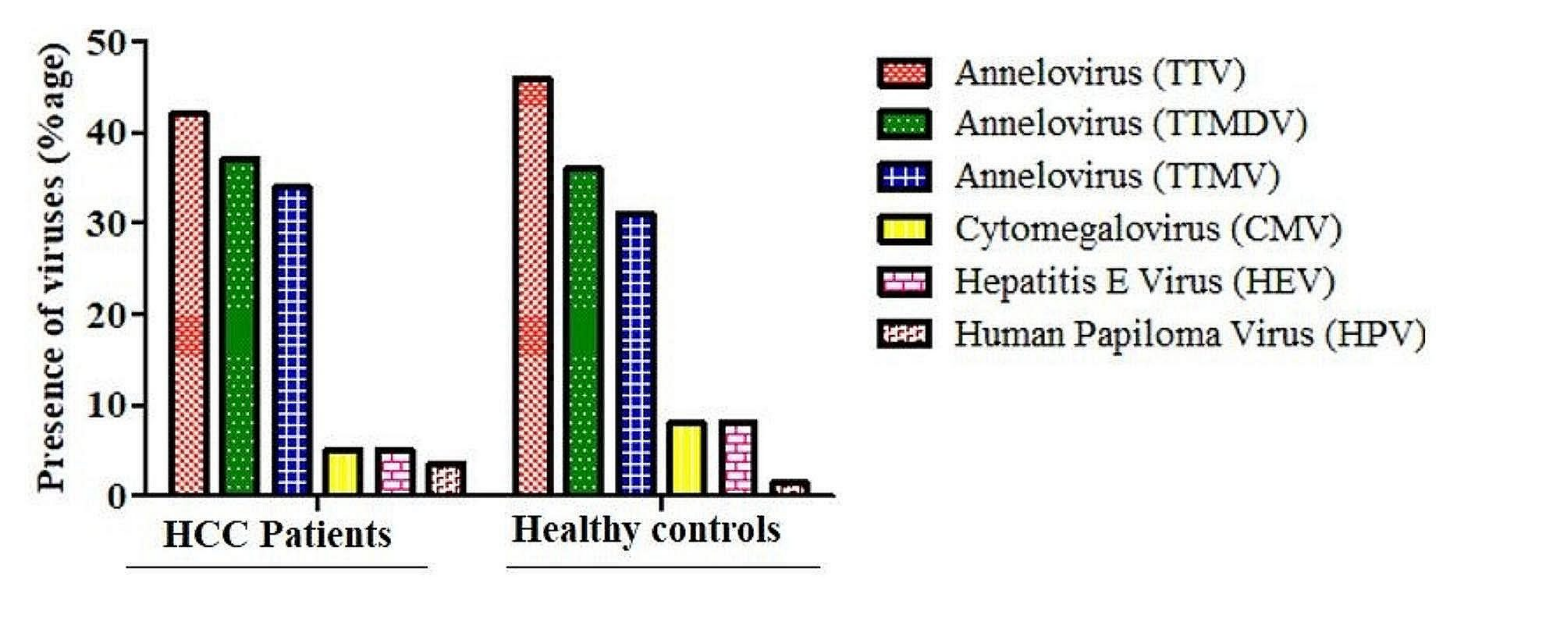
The graph shows the prevalence of HEV, Herpes and Anelloviruses Pooled Samples. Red dotted bar denotes anneloviruses [TTV] variants, green dotted bar represents anneloviruses [TTMDV] variants, blue dotted bar denotes anneloviruses [TTMV] variants, yellow dotted bar denotes[CMV], purple red bar shows hepatitis E virus [HEV], whereas light red bar denotes human papilloma virus [HPV]

The co-infection of TTMV was found in 70% of the samples whereas (Fig. 3). Healthy control group, showed the presence of only TTV (2.3%).

### Detection of HEV, HSV and anelloviruses

Molecular characterization of Herpes viruses (HSV, CMV, VZV, EBV) among HCC patients showed that out of total samples, HEV and CMV were found in 5% of HCC patients indicating presence of viral infection among them. Other viruses including HPV were of 3.5% in prevalence in HCC patients (Fig. 3).

## Discussion

Globally, Hepatocellular Carcinoma (HCC) is the 3rd most frequent cause of cancer-associated mortality being the 7th leading cause of cancer in female and 5th in male and remains dominant in south-east and east Asia (Torre et al. 2015) because of high prevalence of HBV and HCV infection. A total of 381 HCC patients were studied in the current study where male (72.4%) dominating over females as described by other studies earlier (Bray et al. 2018). It could be proposed that androgens have a tumorigenic impact; nevertheless, the precise mechanism remains unexplored (Abbasi et al. 2010). Globally, according to a comprehensive study, both HBV and HCV were the topmost causes of HCC (de Martel et al. 2015). Data reported from Pakistan showed that since 1987 till 2010 approximately 50–60% followed by 20–30% of cases of HCC were infected by HCV and HBV respectively (de Martel et al. 2015). Similarly, in the current study on HCC patients we noted that HCV was most prevalently observed (76.4%) followed by HBV infected (11.5%) as reported similarly from Pakistan (Shehzad et al. 2019). In most of the cases the HCC is associated with the infective agents including HBV and HCV and considered as preventable where in USA it’s incidence become almost tripled between 1975 and 2011 because of high intravenous drugs abusers and increased prevalence of HCV during 1960s and 1970s (Torre et al. 2015). Concurrently, some studies in high risk area of other countries like Japan, China and Taiwan reported the decreasing incidence of HCC because of applying sufficient measures and vaccination against HCV and HBV (Torre et al. 2015). Besides, coinfection with HCV, HBV and HIV, diabetes mellitus, obesity and use of alcohol and lack of mass level awareness are important risk factors for the development of HCC (Mittal et al. 2016; Umer and Iqbal 2016). These studies indicate that by applying appropriate preventive measures like Proper sterilization and avoiding reuse of medical devices for the control of viral agents, mass level awareness, development of competent surveillance measures for early stage diagnosis of HCC and anti-hepatotropic vaccinations, HCC might be controlled/prevented (Kaya et al. 2022; Umer and Iqbal 2016).

The current study also observed that 43% of our populations had single solitary lesion followed by 40.2% with 2–3 solitary lesion as somewhere already reported from the adjacent area of Pakistan (Shehzad et al. 2019). Similarly, in most of the patients (35.9%) the tumor size was 3–5 cm followed by 5–10 cm which is comparable with different previous studies (Curley et al. 2015; Shehzad et al. 2019). The possible reason for higher detection of more than one lesion might be the absence of fully trained sonologist (Curley et al. 2015) as well as the absence of an acute onset and silent progression of the disease till the development of enough large size lesion for diagnosis in Pakistan (Shehzad et al. 2019; Curley et al. 2015). Furthermore, the patients didn’t express any type of special signs and symptoms and their daily activities performance are either normal or mild difficult in general. Common symptoms includes fever, loss of weight, general weakness, abdominal swelling, yellowing of the sclera and skin and right hypochondrium pain as observed in general liver diseases (Poon et al. 2001). Barcelona Clinic Liver Cancer (BCLC) which describe the patient’s performance status, Child Pugh (liver functional status) and stage of cancer including okuda staging (Stage A, B and C), portal invasion, size and number of lesion play an important role in grading of HCC (Pons et al. 2005; Bray et al. 2018). A recent report from Pakistan reported a high percentage of individual with BCLC-B stage (Shehzad et al. 2019) which supported our results of the current study that most of the patients were diagnosed with BCLC-B stage; an intermediate, mostly asymptomatic, with good performance status and multinodular HCC (Butt et al. 2012). Similarly, BCLC-C patients were the second most widely found patients in this study as reported somewhere else (Shehzad et al. 2019) which include symptomatic patients with advanced disease containing extrahepatic spread and vascular invasion (Raza and Sood 2014). According to a study, 70% of the HCC patients are reported in advanced stages (BCLC-B, C, D) in Pakistan (Shehzad et al. 2019) where curative treatments could not be done. Most of the current studied HCC patients have AFP level < 400 as already reported by somewhere else (Shehzad et al. 2019) and therefore, the updated guidelines for the surveillance of HCC recommend ultrasound every 6 months, rather than combination of ultrasonography and AFP levels (Colombo and Sirlin 2019).

The current study observed Torquetino Virus (TTV) in 42.2% of HCV-associated and 31.5% of HBV associated HCC patients. Similarly, the presence of TTV among HCC patients (Pineau et al. 2000; Tagger et al. 1999), HBV and HCV associated HCC patients (Yamamoto et al. 1998), HBV carrier (Devalle and Niel 2004), among non-HCC and HCC patients (Devalle and Niel 2004, 2005; Shibayama et al. 2001), renal transplant patients (Takemoto et al. 2015), lung cancer patients (Bando et al. 2008; Stefani et al. 2021), transplant patients (Focosi et al. 2014; Frye et al. 2019; Gilles et al. 2017; Görzer et al. 2015), HCV patients (Maggi et al. 2003; Mousavi-Nasab et al. 2013; Koohi et al. 2012) has been reported. Furthermore, high load of TTV was non-significantly associated with HCV-associated HCC patients (Tokita et al. 2002) and decompensated cirrhosis or HCC associated HCV patients (Zein et al. 1999). Additionally, TTV-associated HCV-linked HCC patients had higher levels of irregular hepatocytes regeneration (a major risk factor in HCC development) as compare to non-infected TTV patients (Moriyama et al. 2001), aiding the hypothesis about the role of TTV in hepatocarcinogenesis. However, the significant association of HCC with high burden of TTV has been explained through an alternative way by some other studies (Tokita et al. 2002).

The current study also found the prevalence of SEN Viruses in the plasma of HCC patients which is in accordance to the previous report of high prevalence of SEN-V in hepatic viral infected patients from China (Moriyama et al. 2003; Umemura et al. 2001; Shibata et al. 2001). These studies find non-significant adverse clinical characteristics among SEN-V DNA-negative and –positive patients which suggests that co-infection of chronic SEN-V with HCV or HBV associated chronic liver infections didn’t modify the serological features of the patients (Moriyama et al. 2003). The SEN-V has been transmitted through intravenous route, fecal-oral route (Tanaka et al. 2001; Umemura et al. 2001) and vertical infection and henceforth, vertical route and fecal-oral route are thought to be vital for infection with SEN-V transmission as both SEN-V and TTV belongs to the same virus family (Tanaka et al. 2001; Okamoto et al. 1998; Umemura et al. 2001).

The finding of the current study regarding the presence of Herpes viruses showed the prevalence of HSV among HCC patients where several other studies reported its presence among blood donors (Juhl et al. 2010), HIV patients (Zuckerman et al. 2007; Sudenga et al. 2012), central nervous system (Duarte et al. 2019) and general population (Xu et al. 2006) has been already reported in earlier literature. Surprisingly we didn’t detect the presence of EBV DNA among HCC patients as reported in the earlier studies (Gomaa et al. 2022; Li et al. 2004; Chu et al. 2001a; Chen et al. 2019; Akhter et al. 2003; Junying et al. 2003; Zur Hausen et al. 2003; Herrmann and Niedobitek 2003). The current study didn’t find EBV among the plasma of HCC whereas several previous reports published controversial results about the association of EBV with the initiation and development of malignancies like esophageal cancer (Kijima et al. 2001; Wang et al. 1999), breast cancer (Bonnet et al. 1999; Touitou et al. 2001; Chu et al. 2001b; Preciado 2003), colorectal cancer and gastric cancer whereas a strong association has been observed in various studies from Taiwan and Japan as compare to western countries. It has been found in a study that development of HCC has been promoted by infection of EBV, playing a significant role in malignancy of HCC, probably by directly influencing tumorigenic potential or promoting the proliferation of carcinoma cells, exacerbating inflammatory processes in liver tissue, or promoting HCV replication (Li et al. 2004). Henceforth, this study assumes that the association of HCC with EBV may differ with racial and regional differences which need further research.

The current study reported a low prevalence of HEV among the plasma of HCC patients as reported somewhere else (Lin et al. 2020; Atsama et al. 2017; Bai et al. 2018). Additionally, other studies reported that HEV infection accelerate the development of liver cirrhosis (Gérolami et al. 2008; Kamar et al. 2017) which further leads to hepatocellular carcinoma. Furthermore, in case of HBV/HCV or alcohol associated HCC the HEV may work as a co-factor where it can be treated in three months in 80% of the patients by prescribing ribavirin or by decreasing the dosage of immunosuppressive medicine and in case of relapsing or non-responsive virological response, an additional 6-month therapy has been suggested (Kamar et al. 2017). Conclusively, the prevalence of HEV has been neglected in most of the cases of chronic and acute hepatitis as well as HCC (Colson and Raoult 2017). Human cytomegalovirus (HCMV) is a beta herpesvirus which is highly prevalent and transmissible (Britt 2008; Staras et al. 2006) persistently observed in diverse number of body tissues persisting their through hypothesized pathway including latent infection with periodic subclinical reactivation or productive chronic infection and hence never cleared from the body (Coaquette et al. 2004; Britt 2008). The current study finds the prevalence of HCMV among HCC patients as reported somewhere else (Lepiller et al. 2011). Recent research reported the association of HCMV with the inflammatory components of various chronic diseases including cancer (Michaelis et al. 2009), functional impairment (Wang et al. 2010), cognitive decline including vascular dementia (Aiello et al. 2006), cardiovascular disease (Smieja et al. 2003) and hepatitis (Ten Napel et al. 1984). The presence of CMV among HCC patients in the current study might be justified as HCMV could be due to the circulating monocytes reached to liver due to its inflammation or infection of liver vessels (Khan et al. 2009; Guetta et al. 1997). Advancement in CMV infection could play a role in organ failure and mortality (Rafailidis et al. 2008; Razonable and Emery 2004). Concurrently, a recent reports published the potential oncolytic activity, accelerates apoptosis and infiltration and stimulation of immune cell in the microenvironment of tumor (Herbein and Nehme 2020). The anti-cancer abilities of CMV has been proven in various animal and human models of cancers recently (Herbein and Nehme 2020; Klyushnenkova et al. 2012; Kumar et al. 2016; Qiu et al. 2015). The current study established that HCC patients are reservoir of various pathogenic and non-pathogenic viruses which might be involved in pathogenesis, progression and severity of the disease.

### Electronic supplementary material

Below is the link to the electronic supplementary material.

Supplementary Material 1

## Data Availability

All pertinent study data that has been gathered is presented and included in this publication; however, the correspondence author can be contacted with any additional questions.
