# Supplementary material for: Molecular characterization of plasma virome of hepatocellular carcinoma (HCC) patients
Source: AMB Express. 2024 Apr 25;14:46. doi: 10.1186/s13568-024-01696-2 (PMC11045709; doi:10.1186/s13568-024-01696-2)

**Supplementary Table 1.**

**S1 Table: Primer List for the detection of Anelloviruses**

| **S.no** | **Primer name** | **Sequence** | **Melting temperature** |
| --- | --- | --- | --- |
| 1. | SENV AI-1F    AI-1R | 5-TWCYCMAACGACCAGCTAGACCT-3 W = A or T, Y = C or T, M = A or C)  5’- GTT TGT GGT GAG CAG AAC GGA-3’ | 58 |
| 2. | SENV- D-1148F  D-1341R | 5-CTAAGCAGCCCTAACACTCATCCAG-3  5GCAGTTGACCGCAAAGTTACAAGAG-3 | 56 |
| 3. | SENV H-1020F:  H-1138R | 5-TTTGGCTGCACCTTCTGGTT-3  5-AGAAATGATGGGTGAGTGTTAGGG-3 | 58 |

**S2 Table: Primer List for the detection of Senviruses**


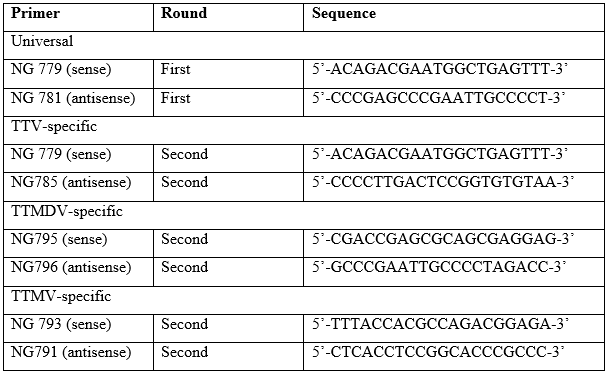


**S3 Table: Primer List for the detection of Herpes Simplex Viruses(HSV), Venzuella Viruses (VZV), Epstein Bar Visues (EBV) and Cytomegaloviruses (CMV)**


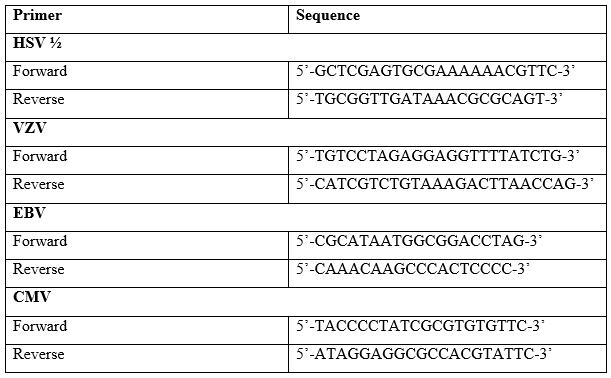

Supplement: Supplementary file 1 — Supplementary Material 1 [file 13568_2024_1696_MOESM1_ESM.docx]
